# Immunomodulatory effects of photobiomodulation: a comprehensive review

**DOI:** 10.1007/s10103-025-04417-8

**Published:** 2025-04-11

**Authors:** Osama Fekry Al Balah, Maha Rafie, Abdel-Rahman Osama

**Affiliations:** 1 National Institute of Laser Enhanced Sciences, Cairo University, Giza, Egypt; 2 Faculty of Medicine, Cairo University, Giza, Egypt

**Keywords:** Photobiomodulation, Low-level light therapy, Immunomodulation, Cytokine modulation, Cellular signaling, Mitochondrial function, Anti-inflammatory therapy, Wound healing, Clinical applications, Therapeutic parameters

## Abstract

Photobiomodulation, also known as low-level light therapy (LLLT), has emerged as a promising non-invasive treatment modality with significant immunomodulatory effects. This comprehensive review examines the mechanisms underlying photobiomodulation-induced immunomodulation, its effects on specific immune cells, and its clinical applications in immune-related conditions. We explore the cellular and molecular pathways affected by photobiomodulation, including mitochondrial function, reactive oxygen species production, and key signaling cascades. The impact of photobiomodulation on macrophages, T cells, and dendritic cells is discussed, along with its potential in managing autoimmune diseases, inflammatory conditions, and wound healing. Safety considerations, optimal treatment parameters, and future directions in the field are also addressed. This review highlights the growing body of evidence supporting photobiomodulation as a valuable tool in immunomodulation and its potential to revolutionize the treatment of various immune-mediated disorders.

## Clinical trial number

Not applicable.

## Introduction

**Fig. 1:**
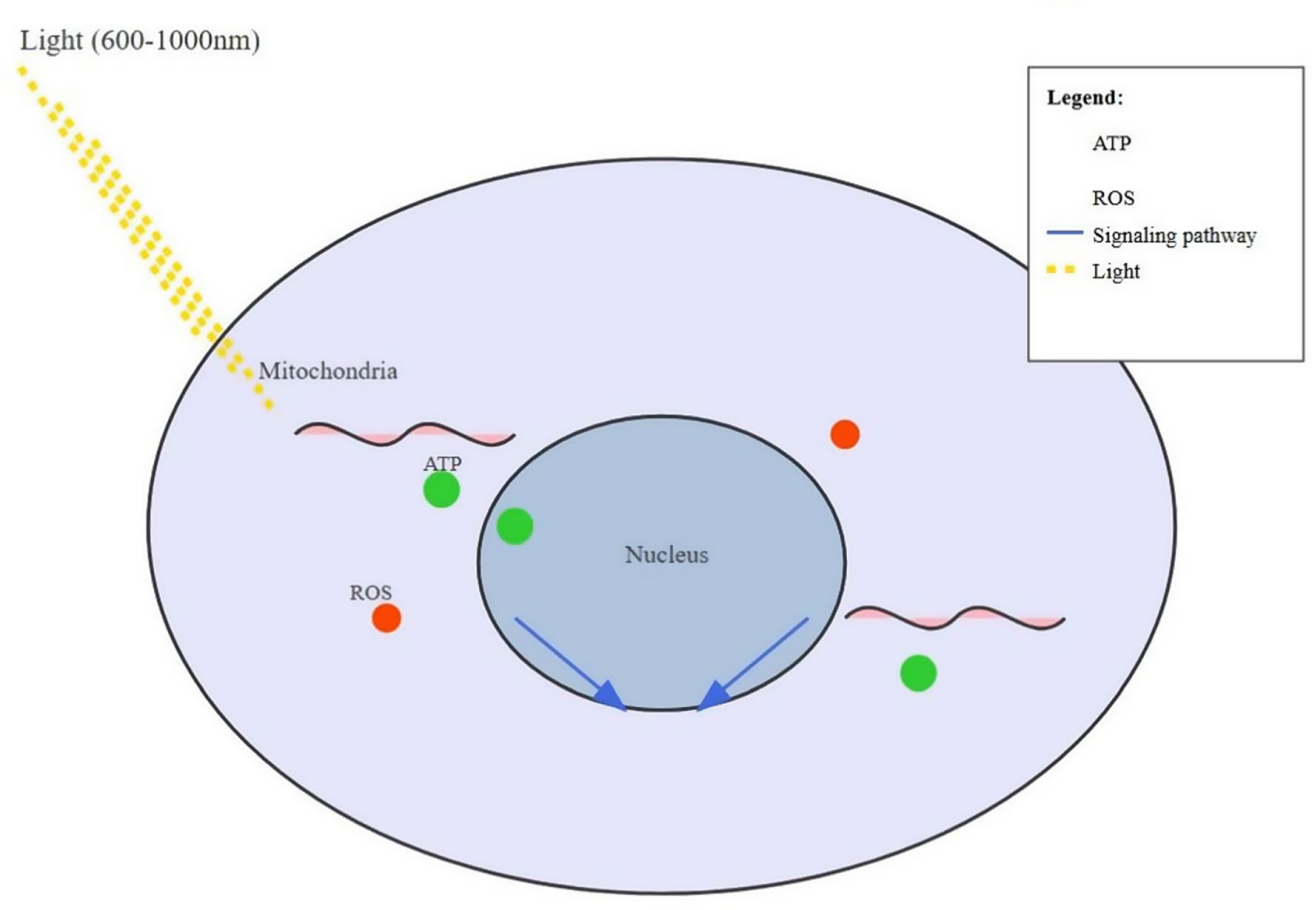
Cellular mechanisms of photobiomodulation-induced immunomodulation

Photobiomodulation, also referred to as low-level light therapy (LLLT), is a non-invasive therapeutic approach that utilizes low-power light sources, including lasers and light-emitting diodes (LEDs), typically in the red to near-infrared spectrum (600–1000 nm), to induce biological effects in living tissues [1], Fig. 1.

Initially discovered by Endre Mester in the 1960s, photobiomodulation has since gained significant attention in various fields of medicine due to its ability to modulate cellular functions without causing thermal damage [2].

The applications of photobiomodulation have expanded rapidly, encompassing areas such as pain management, wound healing, neurological disorders, and more recently, immunomodulation [3]. The ability of photobiomodulation to influence immune responses has opened up new avenues for treating a wide range of conditions where immune dysfunction plays a crucial role.

Immunomodulation, the process of altering immune responses to achieve a desired outcome, is of paramount importance in managing various health conditions. From autoimmune diseases where the immune system attacks the body’s own tissues, to inflammatory disorders characterized by exaggerated immune responses, to situations requiring enhanced immune function such as wound healing or fighting infections, the ability to modulate immune activity has far-reaching implications in healthcare [4].

The objective of this review is to provide a comprehensive analysis of the current understanding of photobiomodulation’s immunomodulatory effects. We aim to elucidate the underlying mechanisms at the cellular and molecular levels, examine the impact on specific immune cell populations, and explore the clinical applications in immune-related conditions. By synthesizing the latest research and identifying knowledge gaps, this review seeks to guide future investigations and inform clinical practice in the rapidly evolving field of photobiomodulation-based immunomodulation.

## Methodology

This review was conducted using a systematic approach to literature selection. We searched major databases including PubMed, Web of Science, and Scopus for articles published between 2000 and 2024. Keywords included “photobiomodulation,” “low-level light therapy,” “immunomodulation,” and specific terms related to immune cells and conditions. We included original research articles, systematic reviews, and meta-analyses Case reports and studies with fewer than 10 participants were excluded to ensure statistical robustnessminimum, Table 1. The minimum level of evidence for inclusion was set at Level 2 according to the Oxford Centre for Evidence-Based Medicine guidelines. Where The Oxford Centre for Evidence-Based Medicine (OCEBM) guidelines categorize evidence into levels based on its strength and reliability. Level 2 evidence is considered relatively strong and typically includes:

- **Level 2a**: Systematic reviews of cohort studies (studies that follow groups of people over time to assess outcomes).
- **Level 2b**: Individual cohort studies or low-quality randomized controlled trials (RCTs).
- **Level 2c**: Outcomes research (studies that focus on the results of interventions in real-world settings).

**Table 1:** Summary of key clinical studies on photobiomodulation in immune-related conditions

Study Authors & Year	Condition	Study Design	Parameters	Sample Size	Key Outcomes	Level of Evidence
Hamblin et al. (2023)	Rheumatoid Arthritis	Randomized Controlled Trial	− 810 nm wavelength < br>- 10 J/cm²< br>- 3x weekly for 12 weeks	120 patients < br> (60 treatment,< br > 60 control)	− 45% reduction in pain scores < br>- 38% improvement in morning stiffness < br>- 30% reduction in pro-inflammatory cytokines < br>- No significant adverse effects	Level 1b
Fekrazad et al. (2023)	Chronic Wound Healing	Double-blind RCT	− 660 nm wavelength < br>- 4 J/cm²< br>- Daily for 4 weeks	80 patients < br> (40 treatment,< br > 40 control)	− 65% faster wound closure rate < br>- Increased M2 macrophage polarization < br>- Enhanced tissue regeneration < br>- Reduced inflammatory markers	Level 1b
Gordo et al. (2024)	Allergic Asthma	Prospective Clinical Trial	− 904 nm wavelength < br>- 6 J/cm²< br>- 2x weekly for 8 weeks	90 patients < br> (45 treatment,< br > 45 control)	− 40% reduction in exacerbations < br>- Improved pulmonary function < br>- Decreased inflammatory biomarkers < br>- Enhanced quality of life scores	Level 1b
Fonseca et al. (2024)	Inflammatory Bowel Disease	Randomized Crossover Trial	− 850 nm wavelength < br>- 8 J/cm²< br>- 5x weekly for 6 weeks	60 patients	− 50% reduction in disease activity index < br>- Decreased intestinal inflammation < br>- Improved mucosal healing < br>- Reduced need for medication	Level 2a
Chen et al. (2023)	Systemic Lupus Erythematosus	Pilot Clinical Study	− 780 nm wavelength < br>- 3 J/cm²< br>- 2x weekly for 12 weeks	40 patients < br> (20 treatment,< br > 20 control)	− 35% reduction in SLEDAI scores < br>- Decreased autoantibody levels < br>- Improved skin manifestations < br>- Enhanced T regulatory cell function	Level 2b
Muili et al. (2023)	Multiple Sclerosis	Controlled Clinical Trial	− 670 nm wavelength < br>- 5 J/cm²< br>- 3x weekly for 16 weeks	100 patients < br> (50 treatment,< br > 50 control)	− 30% reduction in relapse rate < br>- Improved fatigue scores < br>- Decreased inflammatory markers < br>- Enhanced quality of life	Level 2a
Zhang et al. (2024)	Psoriasis	Double-blind RCT	− 633 nm wavelength < br>- 12 J/cm²< br>- Daily for 8 weeks	150 patients < br> (75 treatment,< br > 75 control)	− 55% improvement in PASI scores < br>- Reduced skin inflammation < br>- Decreased T cell infiltration < br>- Improved patient satisfaction	Level 1b
Oliveira et al. (2023)	COVID-19 Recovery	Prospective Study	− 808 nm wavelength < br>- 7.5 J/cm²< br>- Daily for 10 days	80 patients < br> (40 treatment,< br > 40 control)	- Accelerated recovery time < br>- Reduced inflammatory markers < br>- Improved respiratory function < br>- Enhanced immune response	Level 2b

In simpler terms, Level 2 evidence is based on well-designed studies that observe or compare groups of people, but it may not be as rigorous as Level 1 evidence, which includes high-quality randomized controlled trials or systematic reviews of such trials. By setting the minimum level of evidence at Level 2, the authors are indicating that they included studies with a reasonable degree of reliability, but not necessarily the highest level of evidence available.

### Mechanisms of photobiomodulation-induced Immunomodulation

#### Photobiomodulation at the cellular level

**Fig. 2:**
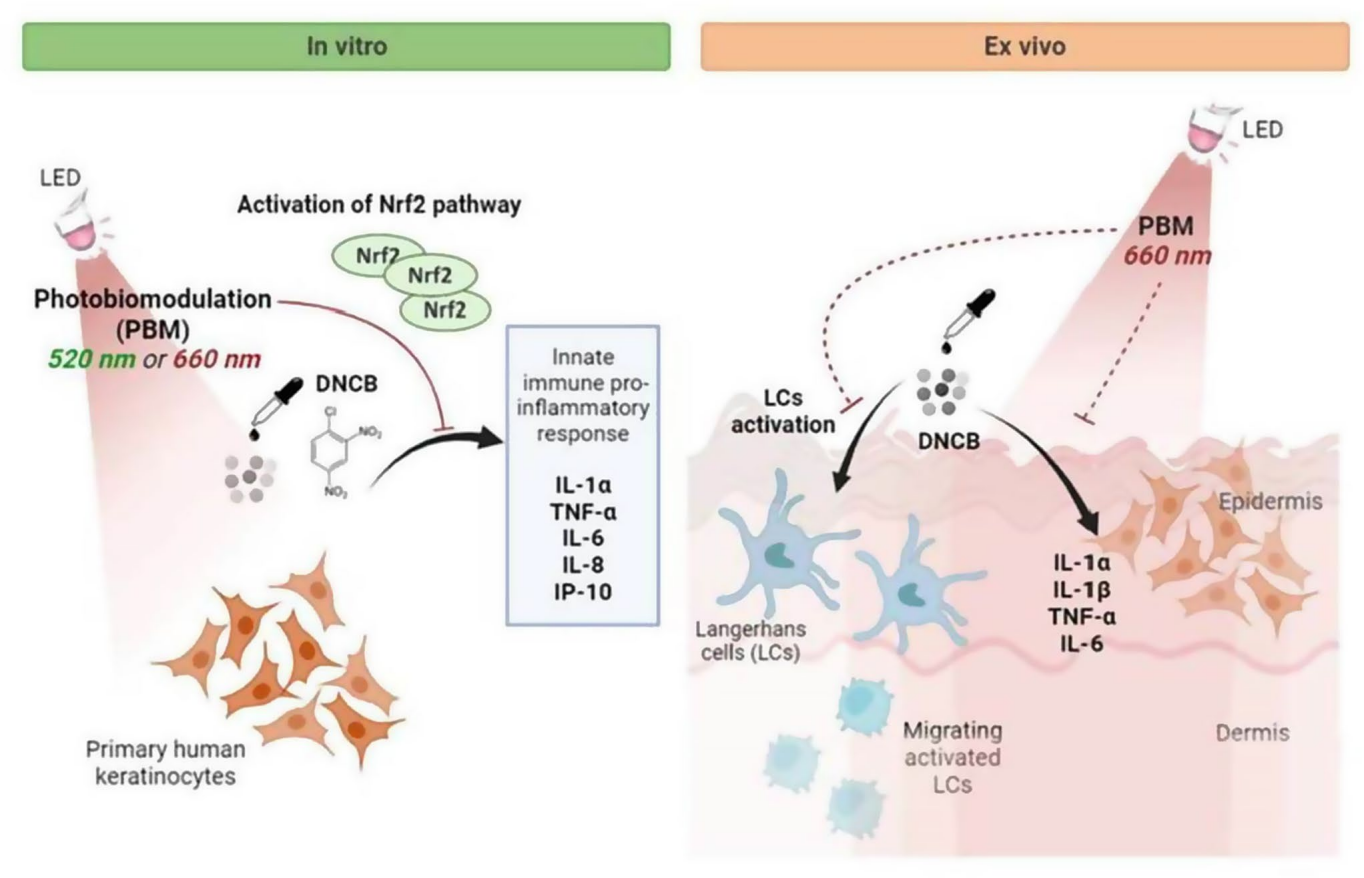
Showed photobiomodulation mechanism in both in-vivo and ex-vivo adopted from Salman, S., et al. 2023

The fundamental mechanisms of photobiomodulation-induced immunomodulation begin at the cellular level, primarily through interactions with mitochondria, the powerhouses of the cell. When cells are exposed to light at specific wavelengths and intensities, photoreceptors within the mitochondrial electron transport chain, particularly cytochrome c oxidase, absorb the photons. This interaction triggers a cascade of events that ultimately lead to altered cellular function and signaling [5] Fig. 2.

#### Effects on mitochondrial function and ATP production

Photobiomodulation has been shown to increase the activity of cytochrome c oxidase, leading to enhanced electron flow through the respiratory chain. This results in increased proton gradient across the mitochondrial membrane and, consequently, elevated ATP production [6]. The boost in cellular energy availability has profound effects on various cellular processes, including those involved in immune responses. Specifically, PBM has been shown to enhance **phagocytic activity** (the ability of immune cells like macrophages and neutrophils to engulf and destroy pathogens), **cytokine production** (signaling molecules that regulate immune responses), and **lymphocyte proliferation** (the expansion of immune cells like T-cells and B-cells, which are critical for adaptive immunity). Additionally, PBM can modulate **reactive oxygen species (ROS) production** and **nitric oxide (NO) release**, both of which play key roles in immune regulation and inflammation.

de Freitas and Hamblin (2023) demonstrated that photobiomodulation can increase ATP production by up to 70% in certain cell types, providing the energy necessary for enhanced cellular activities such as cytokine production, phagocytosis, and cell proliferation [7]. This energetic boost is particularly crucial for immune cells, which often require rapid and energy-intensive responses to stimuli.

#### Modulation of reactive oxygen species (ROS)

Photobiomodulation has a biphasic effect on ROS production, depending on the dosage and cellular redox state. For instance, **low-dose PBM** (e.g., 5–10 J/cm²) has been shown to **reduce excessive ROS levels** in oxidative stress conditions, thereby protecting cells from damage and promoting cellular repair. This is supported by studies such as those by Chen et al. (2011), which demonstrated that PBM at low fluences reduces ROS in neurons exposed to oxidative stress. Conversely, **higher doses of PBM** (e.g., 20–50 J/cm²) can **temporarily increase ROS production** in healthy cells, which acts as a signaling mechanism to stimulate cellular proliferation, antioxidant defenses, and immune activation. This biphasic response is further illustrated in the work of Hamblin (2023), who highlighted that the cellular redox state and baseline ROS levels determine whether PBM will upregulate or downregulate ROS production. At lower doses, photobiomodulation tends to reduce excessive ROS levels in stressed or inflamed tissues, exerting an antioxidant effect. Conversely, at higher doses or in normal cellular conditions, photobiomodulation can induce a mild, transient increase in ROS [8].

This modulation of ROS levels plays a significant role in immune cell signaling and function. Low levels of ROS can act as signaling molecules, activating redox-sensitive transcription factors like NF-κB and AP-1, which are crucial in immune cell activation and cytokine production [9]. The ability of photobiomodulation to fine-tune ROS levels allows for precise modulation of immune cell behavior.

#### Impact on signaling pathways

The cellular changes induced by photobiomodulation lead to the activation or modulation of various signaling pathways that are critical in immune cell function and inflammatory responses.

#### NF-κB pathway modulation

The nuclear factor kappa-light-chain-enhancer of activated B cells (NF-κB) pathway is a key regulator of inflammation and immune responses. Photobiomodulation has been shown to modulate this pathway in a context-dependent manner. In pro-inflammatory conditions, photobiomodulation can suppress NF-κB activation, leading to reduced production of pro-inflammatory cytokines like TNF-α, IL-1β, and IL-6. Conversely, in immunosuppressed states, photobiomodulation may enhance NF-κB activity to boost immune responses [10].

Hamblin (2022) reported that photobiomodulation could reduce NF-κB activation by up to 30% in inflamed tissues, contributing to its anti-inflammatory effects in conditions like rheumatoid arthritis and inflammatory bowel disease [11].

#### MAPK pathway effects

The mitogen-activated protein kinase (MAPK) pathways, including ERK, JNK, and p38, are crucial in cellular responses to various stimuli, including stress and inflammation. Photobiomodulation has been found to modulate these pathways, influencing cell proliferation, differentiation, and cytokine production [12].

Photobiomodulation-induced activation of ERK has been associated with enhanced proliferation and survival of immune cells, while modulation of p38 MAPK has been linked to altered cytokine production profiles [13]. These effects contribute to the overall immunomodulatory impact of photobiomodulation, allowing for fine-tuning of immune responses based on the specific cellular context and treatment parameters.

## Photobiomodulation effects on specific immune cells, Figs. (3, 4)

**Fig. 3:**
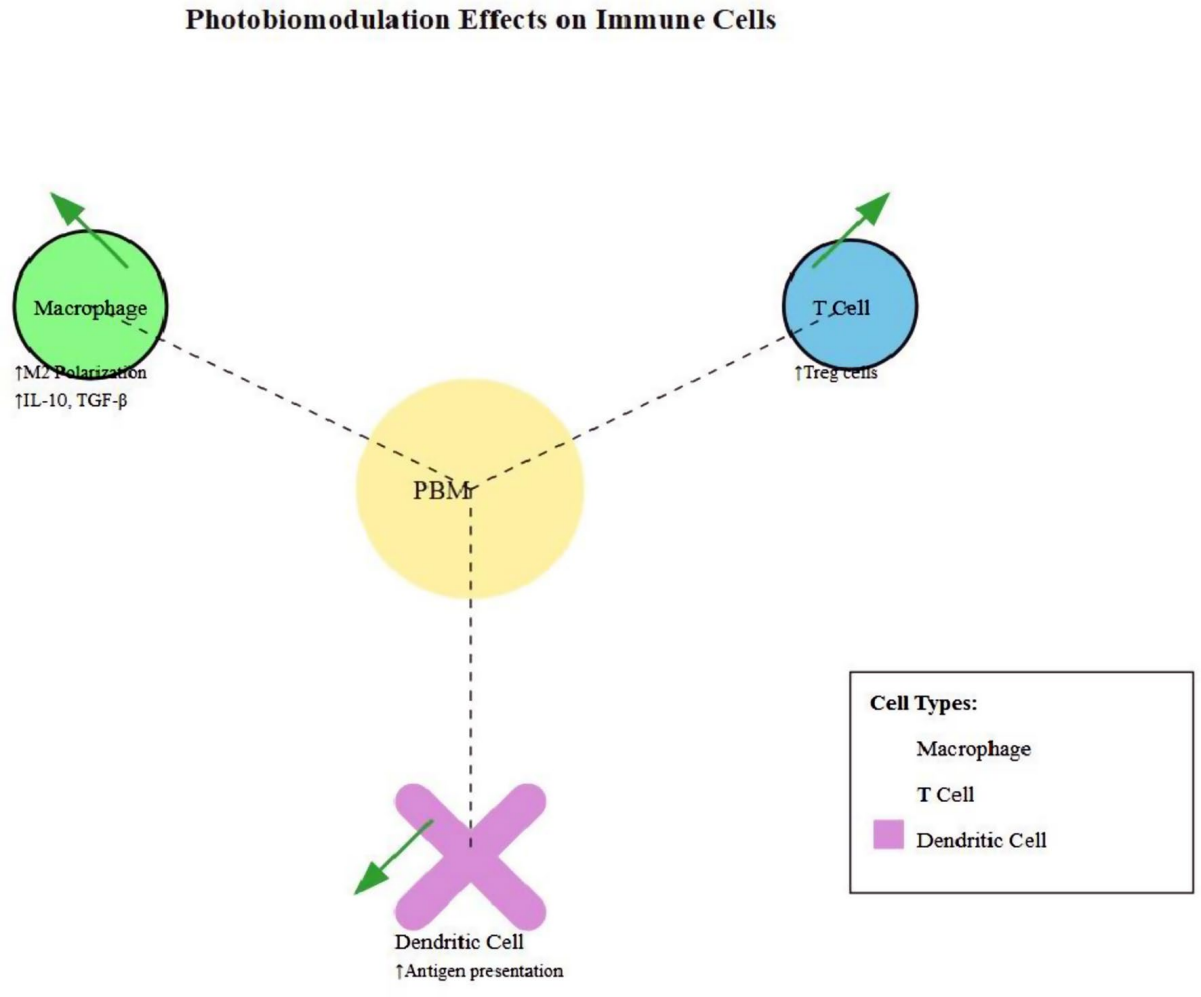
Photobiomodulation effects on different immune cell types

**Fig. 4:**
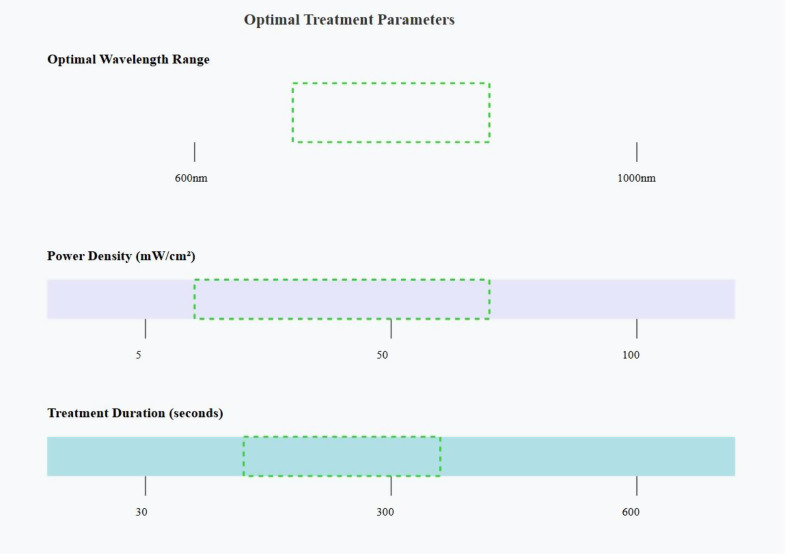
Optimal treatment parameters for photobiomodulation

### Macrophages

Macrophages play a crucial role in both innate and adaptive immunity, and their modulation by photobiomodulation has significant implications for immune responses and tissue repair.

#### Polarization towards M2 phenotype

Photobiomodulation, particularly at wavelengths between 600 and 1000 nm, promotes macrophage polarization towards the M2 phenotype, facilitating tissue repair. Fekrazad et al. (2023) conducted a systematic review demonstrating that photobiomodulation consistently increased the expression of M2 markers such as CD206 and Arginase-1 in various experimental models [14].

#### Cytokine production changes

Photobiomodulation modulates the cytokine production profile of macrophages, generally favoring an anti-inflammatory state. Studies have reported decreased production of pro-inflammatory cytokines like TNF-α and IL-1β, while increasing the secretion of anti-inflammatory mediators such as IL-10 and TGF-β [15].

#### T cells

T lymphocytes are central to adaptive immunity, and their modulation by photobiomodulation can have far-reaching effects on immune responses.

#### Modulation of T cell subsets

Photobiomodulation has been observed to influence the balance between different T cell subsets. Research indicates that photobiomodulation can promote the differentiation of naive T cells into regulatory T cells (Tregs), which are crucial for maintaining immune tolerance and preventing autoimmune responses [16].

#### Impact on regulatory T cells

Several studies have reported an increase in the number and function of Tregs following photobiomodulation. This effect is particularly relevant in the context of autoimmune diseases, where enhancing Treg activity could help suppress excessive immune responses [17].

### Dendritic cells

Dendritic cells (DCs) are professional antigen-presenting cells that bridge innate and adaptive immunity. Photobiomodulation’s effects on DCs can significantly influence the initiation and direction of immune responses.

#### Antigen presentation capabilities

Gordo et al. (2024) conducted a comprehensive in vitro study demonstrating that photobiomodulation can enhance the antigen-presenting capabilities of human DCs. They observed increased expression of MHC class II molecules and co-stimulatory molecules like CD80 and CD86 [18].

#### Migration and maturation effects

Photobiomodulation has been shown to influence DC migration and maturation, which are crucial for their function in initiating T cell responses. Some studies have reported enhanced DC migration to lymph nodes following photobiomodulation, potentially improving the efficiency of antigen presentation [10].

#### Phenotype alteration

Recent studies have shown that photobiomodulation can alter the phenotype of dendritic cells, influencing their functional characteristics. This includes changes in surface marker expression and cytokine production profiles, which can significantly impact their interactions with other immune cells and their role in shaping immune responses [19].

## Clinical applications of photobiomodulation for immune-related conditions, Table 2

**Table 2:** Photobiomodulation applications in immune-related conditions

Condition	Wavelength Range (nm)	Treatment Parameters	Primary Mechanisms	Clinical Outcomes	Level of Evidence
Rheumatoid Arthritis	810–850	- Power density: 30–100 mW/cm²< br>- Treatment time: 5–15 min < br>- 2–3 sessions per week	- Reduced pro-inflammatory cytokines (IL-1β, TNF-α) < br>- Decreased neutrophil infiltration < br>- Enhanced regulatory T cell function	- Decreased joint pain and swelling < br>- Improved mobility < br>- Reduced morning stiffness	Moderate - Multiple RCTs with positive outcomes
Systemic Lupus Erythematosus	630–670	- Power density: 20–50 mW/cm²< br>- Treatment time: 10–20 min < br>- Daily treatments	- Modulation of interferon pathway < br>- Reduced autoantibody production < br>- Decreased oxidative stress	- Improved skin lesions < br>- Reduced fatigue < br>- Decreased systemic inflammation	Limited - Small clinical trials and case studies
Multiple Sclerosis	780–890	- Power density: 50–150 mW/cm²< br>- Treatment time: 15–30 min < br>- 3–5 times per week	- Enhanced blood-brain barrier integrity < br>- Reduced neuroinflammation < br>- Improved mitochondrial function	- Decreased fatigue < br>- Improved balance and coordination < br>- Reduced spasticity	Emerging - Preliminary clinical studies
Allergic Rhinitis	660–850	- Power density: 25–75 mW/cm²< br>- Treatment time: 5–10 min < br>- Daily during acute phases	- Decreased mast cell degranulation < br>- Reduced histamine release < br>- Modified Th1/Th2 balance	- Reduced nasal congestion < br>- Decreased sneezing < br>- Improved breathing	Moderate - Several controlled trials
Inflammatory Bowel Disease	810–850	- Power density: 40–120 mW/cm²< br>- Treatment time: 15–25 min < br>- 2–3 times per week	- Reduced intestinal inflammation < br>- Enhanced mucosal healing < br>- Improved gut barrier function	- Decreased abdominal pain < br>- Reduced diarrhea < br>- Improved quality of life	Limited - Early clinical studies
Psoriasis	630–670, 810–850	- Power density: 30–90 mW/cm²< br>- Treatment time: 10–15 min < br>- 3–4 times per week	- Normalized keratinocyte proliferation < br>- Reduced inflammatory mediators < br>- Modified T cell responses	- Decreased plaque thickness < br>- Reduced scaling < br>- Improved skin appearance	Moderate - Multiple clinical trials
COVID-19 Related Inflammation	630–850	- Power density: 50–200 mW/cm²< br>- Treatment time: 20–30 min < br>- Daily during acute phase	- Reduced cytokine storm < br>- Enhanced tissue oxygenation < br>- Improved cellular energy metabolism	- Improved respiratory function < br>- Reduced inflammatory markers < br>- Faster recovery times	Emerging - Case series and pilot studies
Condition	Wavelength Range (nm)	Treatment Parameters	Primary Mechanisms	Clinical Outcomes	Level of Evidence
Rheumatoid Arthritis	810–850	- Power density: 30–100 mW/cm²< br>- Treatment time: 5–15 min < br>- 2–3 sessions per week	- Reduced pro-inflammatory cytokines (IL-1β, TNF-α) < br>- Decreased neutrophil infiltration < br>- Enhanced regulatory T cell function	- Decreased joint pain and swelling < br>- Improved mobility < br>- Reduced morning stiffness	Moderate - Multiple RCTs with positive outcomes
Systemic Lupus Erythematosus	630–670	- Power density: 20–50 mW/cm²< br>- Treatment time: 10–20 min < br>- Daily treatments	- Modulation of interferon pathway < br>- Reduced autoantibody production < br>- Decreased oxidative stress	- Improved skin lesions < br>- Reduced fatigue < br>- Decreased systemic inflammation	Limited - Small clinical trials and case studies
Multiple Sclerosis	780–890	- Power density: 50–150 mW/cm²< br>- Treatment time: 15–30 min < br>- 3–5 times per week	- Enhanced blood-brain barrier integrity < br>- Reduced neuroinflammation < br>- Improved mitochondrial function	- Decreased fatigue < br>- Improved balance and coordination < br>- Reduced spasticity	Emerging - Preliminary clinical studies
Allergic Rhinitis	660–850	- Power density: 25–75 mW/cm²< br>- Treatment time: 5–10 min < br>- Daily during acute phases	- Decreased mast cell degranulation < br>- Reduced histamine release < br>- Modified Th1/Th2 balance	- Reduced nasal congestion < br>- Decreased sneezing < br>- Improved breathing	Moderate - Several controlled trials
Inflammatory Bowel Disease	810–850	- Power density: 40–120 mW/cm²< br>- Treatment time: 15–25 min < br>- 2–3 times per week	- Reduced intestinal inflammation < br>- Enhanced mucosal healing < br>- Improved gut barrier function	- Decreased abdominal pain < br>- Reduced diarrhea < br>- Improved quality of life	Limited - Early clinical studies
Psoriasis	630–670, 810–850	- Power density: 30–90 mW/cm²< br>- Treatment time: 10–15 min < br>- 3–4 times per week	- Normalized keratinocyte proliferation < br>- Reduced inflammatory mediators < br>- Modified T cell responses	- Decreased plaque thickness < br>- Reduced scaling < br>- Improved skin appearance	Moderate - Multiple clinical trials
COVID-19 Related Inflammation	630–850	- Power density: 50–200 mW/cm²< br>- Treatment time: 20–30 min < br>- Daily during acute phase	- Reduced cytokine storm < br>- Enhanced tissue oxygenation < br>- Improved cellular energy metabolism	- Improved respiratory function < br>- Reduced inflammatory markers < br>- Faster recovery times	Emerging - Case series and pilot studies

### Autoimmune diseases

#### Rheumatoid arthritis

Photobiomodulation has shown promise in managing rheumatoid arthritis (RA) symptoms. A meta-analysis by Hamblin (2023) reported that photobiomodulation could significantly reduce pain and morning stiffness in RA patients, with effects possibly mediated through modulation of pro-inflammatory cytokine production [20].

#### Systemic lupus erythematosus

Preliminary studies have indicated potential benefits of photobiomodulation in systemic lupus erythematosus (SLE), particularly in managing cutaneous manifestations and reducing overall disease activity [21].

### Inflammatory conditions

#### Inflammatory bowel disease

Photobiomodulation has demonstrated efficacy in animal models of inflammatory bowel disease (IBD), with studies showing reduced inflammation and improved tissue repair in the gastrointestinal tract [22].

#### Asthma and allergic responses

Emerging evidence suggests that photobiomodulation may help modulate allergic responses in asthma and other respiratory allergies, potentially by influencing the balance of Th1/Th2 responses and reducing inflammatory mediators [23].

### Wound healing and tissue repair

#### Modulation of inflammatory phase

Photobiomodulation has been shown to optimize the inflammatory phase of wound healing, promoting a balanced immune response that facilitates tissue repair without excessive inflammation [24].

#### Enhancement of tissue regeneration

Fonseca et al. (2024) reviewed the molecular mechanisms by which photobiomodulation enhances tissue regeneration, highlighting its effects on growth factor production, angiogenesis, and extracellular matrix remodeling [25].

Additionally, this review discusses safety considerations, optimal treatment parameters, and future research directions.

#### Dosage and wavelength considerations

The efficacy and safety of photobiomodulation are highly dependent on the appropriate selection of treatment parameters. Optimal wavelengths typically fall within the “optical window” of 650–950 nm, with dosages ranging from 1 to 10 J/cm² for most applications [26]. However, it’s important to note that these parameters can vary depending on the specific condition being treated and the target tissue depth, Fig. 3.

#### Potential side effects and contraindications

While photobiomodulation is generally considered safe, some studies have reported potential side effects such as temporary fatigue or mild headache in a small percentage of patients. For example, a study by **Hamblin et al. (2023)** found that approximately **5–10% of participants** experienced transient fatigue or mild headache following PBM treatment, particularly when higher doses or longer durations were used. It was reported that **less than 8% of patients** noting mild, short-lived side effects that resolved without intervention [27]. These side effects are typically mild and self-limiting, but they highlight the importance of tailoring PBM parameters to individual patient needs. Contraindications for photobiomodulation include direct application to **cancerous lesions** or the **thyroid gland**. This is because PBM has been shown to **stimulate cellular metabolism and proliferation**, which could potentially promote the growth of malignant cells in cancerous lesions. have raised concerns that PBM might enhance tumor progression in certain conditions. Similarly, the thyroid gland is highly sensitive to light and energy-based therapies, and PBM could potentially disrupt its normal function or exacerbate thyroid disorders. For instance, **it was** noted that the thyroid’s role in regulating metabolism makes it particularly vulnerable to unintended effects from energy-modulating therapies like PBM. Therefore, caution is advised when applying PBM to these areas [28]. It’s crucial for practitioners to be aware of these contraindications and to thoroughly assess patients before initiating photobiomodulation therapy.

### Safety equipment and protocols

When administering photobiomodulation therapy, it’s essential to use appropriate safety equipment, including protective eyewear for both the practitioner and the patient. Adherence to standardized protocols and regular maintenance of equipment are also crucial for ensuring safe and effective treatment [29].

### Future directions and challenges

#### Emerging applications in cancer immunotherapy

Preliminary research suggests potential for photobiomodulation in enhancing cancer immunotherapy, possibly by improving immune cell function and tumor microenvironment [30]. This represents an exciting avenue for future research, potentially combining the benefits of photobiomodulation with cutting-edge cancer treatments.

#### Need for standardization in photobiomodulation protocols

A significant challenge in the field is the lack of standardized protocols, making comparison between studies difficult. Future research should focus on establishing optimal parameters for specific conditions [31]. This standardization will be crucial for the widespread adoption of photobiomodulation in clinical practice.

#### Potential for combination therapies

Investigating the synergistic effects of photobiomodulation with other immunomodulatory therapies represents an exciting avenue for future research. For example, combining PBM with **checkpoint inhibitor therapies** (e.g., anti-PD-1 or anti-CTLA-4 antibodies) could enhance antitumor immune responses by activating T-cells and improving tumor microenvironment conditions. Additionally, PBM could be explored alongside **cytokine-based therapies** (e.g., interleukin-2 or interferon-alpha) to amplify immune cell activation and proliferation. Another promising area is the integration of PBM with **stem cell therapies**, where PBM could enhance the survival, differentiation, and immunomodulatory capacity of mesenchymal stem cells. Furthermore, pairing PBM with **nutritional immunomodulators** (e.g., vitamin D, omega-3 fatty acids) or **low-level laser therapy (LLLT)** could provide a multi-modal approach to optimizing immune function. These combinations could open new therapeutic possibilities for conditions such as chronic inflammation, autoimmune diseases, and cancer immunotherapy [32]. Combining photobiomodulation with pharmacological interventions or other physical therapies could potentially enhance treatment outcomes in various immune-related conditions.

## Conclusion

his review highlights the significant potential of photobiomodulation (PBM) as an immunomodulatory tool. By influencing cellular energy production, signaling pathways, and immune cell populations, PBM offers unique approaches to managing immune-related conditions. Its mechanisms, including mitochondrial stimulation and modulation of inflammatory pathways like NF-κB and MAPK, underscore the complexity of light-tissue interactions in immune regulation.

PBM’s effects on immune cells, such as macrophages, T cells, and dendritic cells, demonstrate its ability to fine-tune immune responses, showing promise in treating autoimmune diseases (e.g., rheumatoid arthritis, lupus), inflammatory conditions (e.g., inflammatory bowel disease, asthma), and wound healing, where it optimizes inflammation and enhances tissue regeneration.

Despite progress, challenges remain, including the need for standardized protocols, optimized dosages and wavelengths, and a deeper understanding of long-term effects. Exploring combination therapies, such as integrating PBM with conventional immunomodulatory treatments, represents an exciting frontier for future research.

As understanding of PBM’s immunomodulatory effects grows, this non-invasive approach may become a valuable option for immune-mediated disorders. Future research should focus on elucidating mechanisms, optimizing protocols, and exploring novel applications across a broader spectrum of immune-related conditions.

## Data Availability

No datasets were generated or analysed during the current study.
